# Rationale and design of ON-TRK: a novel prospective non-interventional study in patients with TRK fusion cancer treated with larotrectinib

**DOI:** 10.1186/s12885-022-09687-x

**Published:** 2022-06-07

**Authors:** James C. H. Yang, Marcia S. Brose, Gilberto Castro, Edward S. Kim, Ulrik N. Lassen, Serge Leyvraz, Alberto Pappo, Fernando López-Ríos, John A. Reeves, Marc Fellous, Frédérique Penault-Llorca, Erin R. Rudzinski, Ghazaleh Tabatabai, Gilles Vassal, Alexander Drilon, Jonathan Trent

**Affiliations:** 1 National Taiwan University Cancer Center, Taipei City, Taiwan; 2 Abramson Cancer Center of the University of Pennsylvania School of Medicine, Philadelphia, PA USA; 3 Current affiliation: Sidney Kimmel Cancer Center of Jefferson University Health, Philadelphia, PA USA; 4 Instituto Do Câncer Do Estado de São Paulo, São Paulo, Brazil; 5 Levine Cancer Institute, Atrium Health, Charlotte, NC USA; 6 Current affiliation: City of Hope National Medical Center, Los Angeles, CA USA; 7 Department of Oncology, Rigshospitalet, Copenhagen, Denmark; 8 Charité – Universitätsmedizin Berlin, Berlin, Germany; 9 Department of Oncology, St. Jude Children’s Research Hospital, Memphis, TN USA; 10 Laboratorio de Dianas Terapéuticas, Hospital Universitario HM Sanchinarro, Madrid, Spain; 11 Current affiliation: Department of Pathology, “12 de Octubre” University Hospital, Madrid, Spain; 12 Bayer HealthCare Pharmaceuticals Inc., Whippany, NJ USA; 13 Bayer HealthCare Pharmaceuticals, Inc., Basel, Switzerland; 14 Department of Pathology, Clermont Auvergne University, INSERM U1240 “Molecular Imaging and Theranostic Strategies”, Center Jean Perrin, Montalembert, Clermont-Ferrand, France; 15 Seattle Children’s Hospital and University of Washington Medical Center, Seattle, WA USA; 16 Department of Neurology & Interdisciplinary Neuro-Oncology, University Hospital Tübingen, Hertie Institute for Clinical Brain Research, Tübingen, Germany; 17 Institut Gustave Roussy, Villejuif Cedex, France; 18 Memorial Sloan Kettering Cancer Center, New York, NY USA; 19 Weill Cornell Medical College, New York, NY USA; 20 Sylvester Comprehensive Cancer Center at University of Miami Miller School of Medicine, Miami, FL USA

**Keywords:** Larotrectinib, *NTRK* gene fusions, TRK fusion, Non-interventional study

## Abstract

**Background:**

Tropomyosin receptor kinase (TRK) fusion proteins resulting from neurotrophic tyrosine receptor kinase (*NTRK*) gene fusions are rare primary oncogenic drivers in a wide array of tumors. Larotrectinib is a first-in-class, highly selective, central nervous system-active TRK inhibitor approved by the US Food and Drug Administration (FDA), European Medicines Agency (EMA), and over 40 countries for the treatment of TRK fusion solid tumors in adult and pediatric patients. Due to the rarity of TRK fusion cancer, larotrectinib was granted accelerated approval based on a relatively small number of patients enrolled in three early phase trials. ON-TRK aims to evaluate the safety profile of larotrectinib in a broader population and over extended time periods.

**Methods:**

ON-TRK is a prospective, non-interventional, open-label, multicenter, multi-cohort, post-approval study in adult and pediatric patients with locally advanced or metastatic TRK fusion cancer treated with larotrectinib that will describe the safety and effectiveness of larotrectinib in real-world practice conditions. Adult patients will be grouped by tumor type and followed for at least 2 years. Patients < 18 years old will be enrolled under a ‘pediatric’ cohort regardless of tumor type and will be followed for 5 years to evaluate the risk of potential long-term adverse effects of larotrectinib on their growth and development. The effectiveness of larotrectinib in the overall study population as well as in patient subgroups will also be evaluated. Procedures avoided in patients with infantile fibrosarcoma (e.g., amputation) and the number of patients who were able to undergo surgery with a curative intent (excluding amputation) because of the use of larotrectinib will be described. Larotrectinib treatment patterns in real-world practice, including dosing and duration of treatment, will be described.

**Discussion:**

The FDA Accelerated Approval Program allows for earlier approval of and patient access to drugs that treat serious conditions and fill an unmet medical need. This study is designed to fulfill post-approval requirements set by the FDA as well as post-marketing requirements set forth by local regulatory bodies and is part of the risk management plan for the EMA.

**Study registration:**

This study is registered at ClinicalTrials.gov (NCT04142437).

**Protocol version:**

v2.5, 25 March 2021.

## Background

Tropomyosin receptor kinase (TRK) proteins are encoded by the neurotrophic tyrosine receptor kinase (*NTRK*) genes and mediate neurotrophin signaling, which is central to normal neuronal development and function [1, 2]. Activation of TRK receptors is initiated by the binding of neurotrophins, which causes autophosphorylation of specific tyrosine residues in the activation loop of the kinase domain. This autophosphorylation leads to the phosphorylation of additional tyrosine residues, which allows the docking of cytoplasmic adaptors and enzymes, in turn driving various downstream signaling pathways and subsequent physiological effects on cellular proliferation, growth, differentiation, and survival [2–5].

TRK fusion proteins are the product of *NTRK* gene fusions and have been identified as primary oncogenic drivers in a wide array of tumors [2, 6–8]. *NTRK* gene fusions occur when the 3′ region of the *NTRK* gene is joined with the 5′ end of a fusion partner gene through intra- or interchromosomal rearrangement. This *NTRK* gene fusion encodes a TRK fusion protein that contains the intact catalytic tyrosine kinase domain from the *NTRK* gene as well as one or more dimerization domains from the partner gene. The result is a TRK fusion protein that is constitutively activated, leading to uninterrupted downstream signaling messages that confer oncogenic potential [2, 9].

*NTRK* gene fusions are rare (occurring in < 1% of cancers) but are present in many types of adult and pediatric solid tumors. *NTRK* gene fusions occur more frequently in some rare tumors (e.g., secretory carcinoma of the salivary gland and infantile fibrosarcoma), and less frequently in other more common cancers (e.g., lung cancer and colorectal cancer) [10–13] (Table 1).

**Table 1:** Prevalence of *NTRK* gene fusions in solid tumors

Tumor type	Prevalence	References
Appendiceal	0.56%–2%	Braghiroli et al. 2016 [36]; Forsythe et al. 2020 [37]
Cholangiocarcinoma	4%	Ross et al. 2014 [38]
Colorectal	0.2%–0.5%	Gatalica et al. 2019 [7]; Creancier et al. 2015 [39]; Forsythe et al. 2020 [37]
Glioblastoma	3%	Zheng et al. 2014 [40]
High-grade glioma	21.21%	Forsythe et al. 2020 [37]
High-grade glioma, pediatric	5.3%–6.19%	Forsythe et al. 2020 [37]; Okamura et al. 2018 [41]
Head and neck cancer	0.5%	Stransky et al. 2014 [12]
Infantile fibrosarcoma	70%–91%	Amatu et al. 2019 [2]; Vaishnavi et al. 2015 [9]; Forsythe et al. 2020 [37]
Low-grade glioma	0.4%–0.88%	Stransky et al. 2014 [12]; Forsythe et al. 2020 [37]
Low-grade glioma, pediatric	1.61%	Forsythe et al. 2020 [37]
Lung adenocarcinoma	0.1%	Gatalica et al. 2019 [7]; Farago et al. 2015 [42]; Forsythe et al. 2020 [37]
Melanoma	0.3%	Stransky et al. 2014 [12]; Forsythe et al. 2020 [37]
Papillary thyroid carcinoma	< 5%–25%	Brzezianska et al. 2006 [43]; Greco et al. 2010 [44]; Wajjwalku et al. 1992 [45]; Said et al. 1994 [46]; Butti 1995 [47]; Delvincourt et al. 1996 [48]; Bounacer et al. 2000 [49]; Forsythe et al. 2020 [37]
Pilocytic astrocytoma	3%	Jones et al. 2013 [50]
Secretory breast carcinoma	92.87%	Tognon et al. 2002 [51]; Forsythe et al. 2020 [37]
Secretory carcinoma of salivary gland	66%–93%	Skalova et al. 2014 [52]; Skalova et al. 2010 [53]; Forsythe et al. 2020 [37]
Soft tissue sarcoma (non-infantile fibrosarcoma)	< 5%	Demetri et al. 2020 [55]
Spitzoid melanoma	16%	Wiesner et al. 2014 [54]

Testing for *NTRK* gene fusions should be considered part of the standard diagnostic work-up for solid tumors in order to optimize treatment, as there are approved small-molecule inhibitors with activity against TRK fusion cancer [14, 15]. Fluorescence in situ hybridization (FISH), reverse-transcription polymerase chain reaction (RT-PCR), or next-generation sequencing (NGS) are the recommended testing methods to detect the presence of an *NTRK* gene fusion [16, 17]. While pan-TRK immunohistochemistry (IHC) is more cost- and resource-efficient than these molecular testing methods, a positive IHC test is insufficient to confirm a diagnosis of TRK fusion cancer. Moreover, cancers involving tissues with endogenous basal wild-type TRK protein expression, such as central nervous system (CNS), endocrine, and smooth muscle tumors, can produce false positive results. Therefore, it is necessary to confirm any positive IHC results with molecular testing [18, 19]. Given the relatively small number of pediatric patients with cancer as compared with adult patients, it may be feasible for pediatric patients to undergo molecular testing upon diagnosis, particularly with tumor types that are known to harbor *NTRK* gene fusions. For instance, the *ETV6-NTRK3* gene fusion is pathognomonic for infantile fibrosarcoma and is routinely detected by break-apart FISH at diagnosis [19].

Larotrectinib is a first-in-class, highly selective, CNS-active oral TRK inhibitor with low nanomolar potency against all three TRK family members (TRKA, TRKB, and TRKC) [6]. Larotrectinib potently inhibits adenosine triphosphate binding to the TRKA, TRKB, and TRKC catalytic domains, while showing low binding affinity for other tyrosine kinases [13, 20]. A primary safety and efficacy analysis used pooled data from the first 55 patients with advanced progressive cancers harboring *NTRK* gene fusions who enrolled in three phase I and II trials (NCT02122913, NCT02637687, and NCT02576431), with 17 unique cancer diagnoses included. The objective response rate (ORR) assessed by an independent review committee (IRC) was 75% (complete response rate of 13%). Efficacy was observed regardless of tumor type or patient age, *NTRK* gene, or fusion partner. At 1 year, 71% of responses were ongoing and 55% of patients remained free of progression. In this primary analysis population, larotrectinib demonstrated a low frequency of clinically significant adverse events (AEs) in both adult and pediatric patients with TRK fusion cancer; 93% of AEs reported were grade 1–2. The most common grade 3–4 AEs were anemia (11%), increased aspartate aminotransferase or alanine aminotransferase (7%), weight increase (7%), and decreased neutrophil count (7%). No grade 3 treatment-related AEs occurred in more than 5% of the patients and there were no grade 4–5 treatment-related AEs [21]. Based on these phase I/II data, larotrectinib became the first tyrosine kinase inhibitor approved by the US Food and Drug Administration (FDA) for tumor-agnostic use in both adult and pediatric patients with advanced TRK fusion solid tumors. It became the first drug granted tumor-agnostic approval by the European Medicines Agency (EMA) and is now approved in over 40 countries for tumor-agnostic use in patients of all ages with TRK fusion cancer [22, 23]. In subsequent data cuts with longer follow-up, overall tumor responses were consistent and safety profiles were comparable with previous reports, demonstrating the durability and long-term safety of larotrectinib [24].

Efficacy of neoadjuvant larotrectinib therapy has also been demonstrated in situations in which the patient would otherwise be subjected to amputations or disfiguring surgeries. Five children with locally advanced TRK fusion sarcomas achieved a partial response and underwent surgical resection after a median of six treatment cycles. Based on institutional pathologic assessment, surgical resections were R0 (negative resection margins with no tumor at the inked resection margin) in three patients, R1 (microscopic residual tumor at the resection margin) in one patient, and R2 (macroscopic residual tumor at the resection margin) in one patient. Three patients achieved complete or near-complete pathological responses and at last follow-up remained disease free 7–15 months after surgery [25, 26].

In an integrated dataset of 159 patients with non-CNS TRK fusion cancer, larotrectinib demonstrated durable efficacy and a favorable long-term safety profile [24]. The most common tumor types in the integrated dataset included soft tissue sarcoma (43%, including infantile fibrosarcoma and gastrointestinal stromal tumor), thyroid cancer (16%), salivary gland cancer (13%), and lung cancer (8%). Larotrectinib showed efficacy regardless of tumor type, with an investigator-assessed ORR of 79%. Median duration of response (DOR) was 35.2 months. Median progression-free survival (PFS) was 28.3 months, and median overall survival (OS) was 44.4 months [24].

No new safety signals for larotrectinib were identified in the expanded safety population (*n* = 260). AEs were primarily of grade 1–2, and the pattern and frequency were similar across age groups. The most frequently occurring AEs of any grade were fatigue (33%), increased alanine aminotransferase (28%), and cough (28%). Grade 3–4 larotrectinib-related AEs were reported in 13% and 1% of patients, respectively. The most common were increased alanine aminotransferase (3%), anemia (2%), and decreased neutrophil count (2%) [24].

Patient-reported outcomes also showed improvement in patients treated with larotrectinib; rapid, sustained, and clinically meaningful improvements in quality of life were observed in the majority of patients [27]. Larotrectinib has also demonstrated intracranial activity in patients with primary CNS tumors [28] or non-CNS solid tumors with brain metastases [24].

Results from these clinical trials demonstrated that in addition to having tumor-agnostic activity, larotrectinib is also effective across age groups, including in children and adolescents. Of the 159 patients analyzed in the integrated dataset, 52 (33%) were aged < 18 years old [29]. Tumor types included infantile fibrosarcoma (56%), other soft tissue sarcoma (37%), thyroid (4%), congenital mesoblastic nephroma (2%), and melanoma (2%). ORR was 92%; at the time of data analysis, medians for DOR, PFS, and OS were not reached. With the duration of treatment ranging from 1.3 + to 34.0 + months, larotrectinib was well tolerated with low rates of discontinuation due to treatment-related AEs and low rates of neurologic AEs.

Longer-term safety evaluation is required, and this is particularly relevant for pediatric patients in order to investigate potential developmental effects of treatment. There are limited available data on the safety profile of larotrectinib in a broader population and over a longer time period. Real-world safety and effectiveness data in a larger patient population are needed with a longer-term follow-up. The ON-TRK study is a prospective, open-label, multicenter, multi-cohort, non-interventional study in patients with locally advanced or metastatic TRK fusion cancer treated with larotrectinib. The aim of the ON-TRK study is to describe the safety and effectiveness of larotrectinib in this patient population under real-world conditions in standard clinical practice. As it is a post-approval safety study, it is also designed to fulfill FDA requirements for the accelerated approval of larotrectinib, which include additional studies to validate and describe the clinical benefits of larotrectinib. Post-approval commitments set forth in the FDA’s accelerated approval of larotrectinib require enough pediatric patients be followed for 5 years to evaluate the risk of potential long-term adverse effects of larotrectinib on growth and development and the assessment of unexpected risks of serious AEs (SAEs) in patients requiring a third larotrectinib dose modification due to toxicity [30]. This study is also intended to fulfill the post-marketing requirements set forth by the regulatory bodies of Canada and South Korea, and is part of the risk management plan provided to the EMA [31].

## Methods

### Study overview

ON-TRK is an international, prospective, open-label, multicenter, multi-cohort, non-interventional study. The guidelines on good pharmacovigilance practices (GVP module VI [32] and GVP module VIII [33, 34]) will be followed. Before documentation of any patient data, informed consent will be obtained from the patient, parent/legal guardian, or legal representative. The study is registered on ClinicalTrials.gov (NCT04142437).

### Study design

#### Participants

**Fig. 1:**
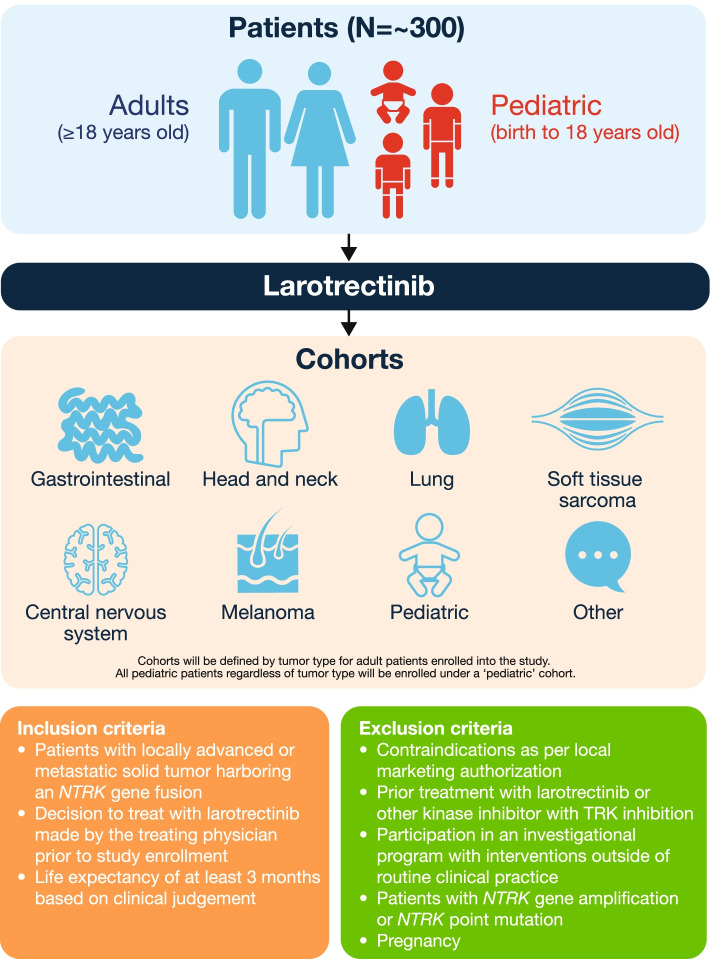
Study design. *NTRK* neurotrophic tyrosine receptor kinase, *TRK* tropomyosin receptor kinase

Inclusion criteria include adult and pediatric (< 18 years old) patients with locally advanced or metastatic solid tumors with an *NTRK* gene fusion for whom a decision to treat with larotrectinib has been made before enrollment in the study (Fig. 1). Patients with prior TRK inhibitor therapy and patients with *NTRK* genomic alterations other than fusions (such as amplifications or point mutations) will be excluded.

*NTRK* gene fusions will be detected locally by NGS, FISH, RT-PCR, or any other genomic test able to detect *NTRK* gene fusions. Cohorts will be defined by the tumor types of enrolled adult patients, including gastrointestinal, head and neck, lung, soft tissue sarcoma, primary CNS, melanoma, or ‘other’. All pediatric patients, regardless of tumor type, will be enrolled within a ‘pediatric’ cohort. Per the study protocol, central pathological review is not planned; all pathology data will be collated locally at each study site. The decision on the dose and duration of treatment is solely at the discretion of the treating physician, based on the recommendations included in the local product information. Examinations and the laboratory monitoring schedule will follow local label recommendations in line with local standard of care.

### Objectives

The study objectives and associated variables are listed in Table 2. Up to 300 patients with *NTRK* gene fusion cancer will be included in this analysis. As the primary objective of this study is to describe the incidence of treatment-emergent AEs (TEAEs) in a real-world setting, the rationale for this study size was based on the probability of observing at least one TEAE event for a range of true incidence rates, including some of the uncommon events. For a true event incidence of 1% and a sample size of 300 patients, the probability of observing at least one event is 95%. Therefore, a total of approximately 300 patients was considered sufficient to observe at least one AE for even uncommon events.

**Table 2:** Study objectives and variables

Objective	Variables
**Primary**: safety of larotrectinib in adult and pediatric patients with locally advanced or metastatic TRK fusion cancer	TEAEs in real-world practice conditions • Incidence • Severity • Seriousness • Outcome • Causality assessment (ARs)
**Secondary**: • Effectiveness of larotrectinib as per investigator assessment in the total study population as well as in patient subgroups (including, but not limited to, age, *NTRK* gene, *NTRK* gene fusion partner, testing methodology, country/region, and prior therapy [type and/or number of lines of therapy])	• Patient demographic and baseline characteristics • Disease history (including pathology report at baseline and subsequently as applicable) • Comorbidities • Method for detecting *NTRK* gene fusion (including testing report at baseline and subsequently as applicable) • ECOG, Lansky, or Karnofsky performance status • Tumor assessments (ORR, DCR, DOR, TTR, PFS, OS) per investigator assessment • Date of radiologic or clinical progression on larotrectinib
• Larotrectinib treatment patterns	• Larotrectinib use (initiation and termination dates, dosage, and dose modification)
• Long-term effects of larotrectinib on growth, developmental milestones, and sexual development in the pediatric cohort • Long-term effects of larotrectinib on neurologic outcomes in all patients	• Developmental milestones; age at adrenarche or menarche (if applicable); Tanner scale • Neurological examination
	• Laboratory examination data; and date of death/last follow-up
**Exploratory:** • Effectiveness of larotrectinib	• Tumor assessments (ORR, DCR, DOR, TTR, PFS, OS) based on radiologic assessments of tumor response per IRC, as applicable
• Procedures avoided because of the use of larotrectinib (e.g., amputation or other disfiguring procedures) in patients with infantile fibrosarcoma	• Whether an amputation was considered for the patient prior to treatment with larotrectinib (e.g., in patients with infantile fibrosarcoma)
• Number of patients who underwent surgery with a curative intent (excluding amputation) because of the use of larotrectinib	• Surgery (with curative intent) while on larotrectinib
• Description of systemic treatments prior to larotrectinib therapy	• Previous systemic therapy (initiation and termination dates, doses, duration of treatment, best tumor response to therapy, date of radiological progression on therapy, reasons for discontinuation)

*AR* Adverse Reaction, *DCR* Disease Control Rate, *DOR* Duration of Response, *ECOG* Eastern Cooperative Oncology Group, *IRC* Independent Review Committee, *NTRK* Neurotrophic Tyrosine Receptor Kinase, *ORR* Objective Response Rate, *OS* Overall Survival, *PFS* Progression-free Survival, *TEAE* Treatment-emergent Adverse Event, *TRK* Tropomyosin Receptor Kinase, *TTR* Time To Response

Secondary objectives include describing the effectiveness of larotrectinib by local investigator assessment, including by subgroups of patients (e.g., age, *NTRK* gene, *NTRK* gene partner, testing methodology, country/region, prior therapy), patterns of larotrectinib treatment, long-term effects of larotrectinib on growth, developmental milestones, and sexual development of the pediatric cohort, and long-term effects of larotrectinib on neurologic outcomes for all patients (Table 2).

### Statistical analyses

Data analyses will be explorative and descriptive in nature. All variables will be analyzed descriptively with appropriate statistical methods: categorical variables by frequency tables (absolute and relative frequencies) and continuous variables by sample statistics (i.e., mean, standard deviation, minimum, median, quartiles, and maximum). Continuous variables will be described by absolute values and as change from baseline per analysis time point, if applicable. Patients who took at least one dose of larotrectinib will be included in the safety analysis set, which will consist of patients who took at least one dose of larotrectinib, did not violate a major inclusion/exclusion criterion, and had at least one post-baseline assessment after receiving larotrectinib. Effectiveness data will be analyzed on the full analysis set. Demographic and baseline data will be described for both full and safety analysis sets. All analyses will be performed for the total study population and by cohorts, as appropriate. Additional analyses may be performed separately for each participating country if patient numbers are sufficient and if required for local reasons. Whenever reasonable, data will be summarized by subgroups (e.g., age, sex, other baseline characteristics). All therapies will be coded using the World Health Organization Drug Global international reference. Any diagnoses and diseases will be coded using the latest Medical Dictionary for Regulatory Activities (MedDRA) version.

#### Analysis of primary variable

AEs will be summarized for the safety population using the latest version of the MedDRA as well as the National Cancer Institute (NCI) Common Terminology Criteria for Adverse Events (CTCAE) v5.0. Incidence proportions will be calculated based on the total number of patients valid for safety and calculated by MedDRA System Organ Class, Preferred Term, and worst CTCAE grade. These analyses will be performed for TEAEs, drug-related TEAEs, treatment-emergent SAEs, drug-related treatment-emergent SAEs, and TEAEs leading to dose reduction, dose interruption, or permanent dose discontinuation. In addition, exposure-adjusted incidence rates will be summarized for TEAEs, defined as the total number of subjects with the AE divided by total subject time at risk in years.

For vital signs, descriptive statistics will be calculated by visit. Summary statistics on change from baseline for laboratory parameters will be reported for each visit. The change from baseline to worst post-baseline value will also be summarized. Where possible, laboratory data will be graded using the mapping provided within the NCI-CTCAE manual version that is valid at the time of analysis. CTCAE severity grading for laboratory abnormalities is mainly based on applicable laboratory threshold values outlined in NCI-CTCAE manuals. Frequency of laboratory abnormalities will be tabulated by NCI-CTCAE category and worst grade as well as by changes in severity from baseline to worst value post-baseline.

#### Analysis of secondary variables

All summaries with respect to effectiveness data will be descriptive. The primary data source for effectiveness endpoints ORR, disease control rate (DCR), DOR, time to response (TTR), and PFS will be based on tumor response as assessed by the investigator for the full analysis set. The estimates of ORR and DCR and the corresponding confidence intervals (CIs) will be provided. For the time-to-event variables DOR, PFS, and OS, the medians and survival rates at various time points with 95% CI will be estimated by the Kaplan–Meier method. TTR will be summarized for responders only using descriptive statistics. Radiologically or clinically documented progression of tumor will be considered as disease progression. Subgroup analyses stratified by prognostic, predictive, or other factors collected at baseline, as mentioned earlier, may be explored.

Duration of larotrectinib treatment is defined as the time (months) from the start of larotrectinib treatment to the day of permanent discontinuation (including death). For larotrectinib treatment, descriptive statistics will be calculated for the treatment duration, starting dose, and average dose. In addition, duration of larotrectinib treatment will be analyzed using a Kaplan–Meier approach, censoring subjects who have ongoing treatment at the time of analysis. The following data will be summarized for larotrectinib treatment: the number of patients with dose modification (e.g., reduction, interruption, re-challenge at protocol dose), number of dose modifications, and frequencies of reasons for dose modifications and permanent discontinuation.

For the pediatric cohort only, change in height and weight from baseline will be summarized at each visit. Age at adrenarche for males and menarche for females will be summarized. In addition, the number and percentage of patients with abnormal Tanner stage, abnormal neurological assessments, and abnormal developmental milestones will be presented.

#### Analysis of exploratory variables

Effectiveness variables, including ORR, DCR, DOR, TTR, and PFS, will also be determined by an IRC, based on radiological assessments of tumor response as applicable. These effectiveness variables will be determined by an IRC for patients who have technically adequate baseline and on-treatment radiological assessments of tumor response that have been centrally collected at the imaging core laboratory. In patients who had systemic anti-cancer therapy prior to the study, the treatment data will be summarized descriptively. Dosing of other treatment used prior to the study, including start/stop date, duration of treatment, given doses, reasons for discontinuation, and time from discontinuation of treatment to start of larotrectinib, will be retrospectively collected and summarized descriptively in the total study population. For patients with infantile fibrosarcoma, the number and percentage of patients who avoided amputation will be reported. The number and percentage of patients who underwent a surgery for a curative intent while being treated with larotrectinib (excluding amputation) will also be presented. In addition, local protocol amendments may be requested. For example, patient-reported outcomes data will be collected in Austria, Germany, and Canada.

#### Timelines and milestones

The recruitment period will be 36 months. The end of the study for all cohorts, except the pediatric cohort, will happen after the final patient has been in the study for at least 24 months or is no longer under observation, owing to being lost to follow-up, withdrawal, or death. For the pediatric cohort, each patient will be followed up for at least 60 months from larotrectinib initiation unless the patient is discontinued due to loss to follow-up, withdrawal, or death.

The study will be closed 60 months after the last pediatric patient has started treatment with larotrectinib or when no patient is still under observation due to loss to follow-up, withdrawal, or death. For all patients still under observation at time of study closure, a last disease status documentation will be required along with the completion of the final information collection.

Safety reviews will be performed annually, with the first review starting 1 year after the first patient is enrolled. Interim reviews for safety and effectiveness will be performed after approximately 50 patients pooled across tumor types complete at least 6 months of treatment or discontinue treatment. Subsequent reviews will be performed after approximately 150 and 300 patients have met the same conditions. In addition, reviews by cohort type will be performed after approximately 10 patients per cohort complete at least 6 months of treatment or discontinue treatment. The various reviews may be combined if they are expected to occur within approximately 1 month of each other.

## Current study status

The ON-TRK study is currently recruiting patients and as of November 2021, 22 patients have been enrolled. The study has an estimated primary completion date of November 2029 and an estimated study completion date of March 2030 [35]. To date, numerous study sites currently recruiting are in Belgium, Canada, Germany, Sweden, Switzerland, and the USA; other study sites and countries will be recruited to participate in the study when local approval and reimbursement for larotrectinib are granted if deemed relevant for the completion of the study (https://clinicaltrials.gov/ct2/show/NCT04142437).

## Discussion

An improved understanding of the mechanisms that drive tumorigenesis has led to substantial advances in the treatment of cancer; however, an unmet need for highly effective and well-tolerated therapies remains, particularly in the metastatic setting and in rare tumors. Identification of recurrent oncogenic drivers has led to a precision medicine approach in which specific genetic alterations are targeted by selective agents. Furthermore, emerging evidence suggests that certain genomic alterations are present as primary oncogenic drivers across a wide variety of different tumor types, providing the rationale for a tumor-agnostic treatment approach. The larotrectinib development program was designed with such an approach, with the aim of demonstrating the activity and safety of larotrectinib in a wide variety of tumor types. As such, no central pathological review is planned given that there is less emphasis on the tumor-specific details.

The FDA Accelerated Approval Program was initiated to allow for earlier approval (and patient access) of drugs that treat serious conditions and fill an unmet medical need, based on a surrogate endpoint. Drugs which receive accelerated approval under this program are required to be further studied in phase IV confirmatory trials.

Larotrectinib was granted accelerated approval based on data from three phase I/II single-arm studies, but safety data are relatively limited, particularly with respect to patient numbers and long-term exposure. ON-TRK is the first prospective, non-interventional study of a TRK inhibitor designed to support the conversion of an accelerated approval to a full approval. The study will provide further evidence to support the safety and effectiveness of larotrectinib in patients with locally advanced or metastatic TRK fusion cancer in real-world practice conditions. Larotrectinib was granted accelerated approval contingent upon fulfilling post-approval requirements in order to obtain full approval. One such requirement is to evaluate an adequate number of patients to characterize response and durability of response for each of the following tumor types: CNS tumors, colorectal cancer, melanoma, and non-small cell lung cancer (NSCLC). A minimum of 40 patients with cancers other than CNS tumors, colorectal cancer, infantile fibrosarcoma, melanoma, NSCLC, salivary cancers, soft tissue sarcoma, and thyroid cancer (e.g., biliary tract cancers, breast cancer, cholangiocarcinoma, gastrointestinal stromal tumors) will also be studied. Evaluating response in these tumor types will further support the tumor-agnostic designation of larotrectinib and may enable additional subgroup analysis by primary tumor type. Another requirement is to conduct a study of larotrectinib following an adequate number of pediatric patients with TRK fusion cancer for 5 years to evaluate the risk of potential long-term adverse effects of larotrectinib on their growth and development; this is covered as a secondary objective of ON-TRK. Patients will be evaluated for age-appropriate growth and developmental milestones and undergo neurological examination at suitable intervals (such as every 6 months) until larotrectinib is discontinued or for at least 5 years, whichever occurs first. In describing AEs requiring dose modifications, ON-TRK will also fulfill another requirement, which is to assess any unexpected risks of SAEs in patients requiring a third dose modification of larotrectinib due to toxicity. An adequate number of adult or pediatric patients with a body surface area of at least 1.0 m^2^ requiring a third dosage modification due to a treatment-related AE will receive larotrectinib 100 mg orally once daily to better describe the tolerability of this approved dosage modification for larotrectinib. The following information will be collected from each patient: age and body surface area (if pediatric), treatment-related AEs leading to the first and second dose reductions of larotrectinib, duration of treatment on prior dose levels, duration of treatment at the 100-mg oral once-daily regimen, best overall response and DOR, and tumor information collected while receiving the 100-mg oral once-daily regimen. An additional request from the FDA was to assess effectiveness by an IRC in this non-interventional study.

Additional trials are ongoing in specific populations of patients with TRK fusion cancer that will gather additional safety and efficacy data for larotrectinib. Recruitment is currently ongoing for the phase II NAVIGATE basket trial (NCT02576431) for patients with kidney cancer, squamous cell cancer of head or neck, ovarian solid tumors, melanoma, non-secretory breast cancer, and colorectal cancer harboring *NTRK* gene fusions. The phase II MATCH screening trial (NCT02465060) is studying the efficacy of various treatments (including larotrectinib) directed by genetic testing work in adult patients with solid tumors or lymphomas with progression following at least one line of standard treatment, or for which there are no approved or standard therapies. The phase II pediatric MATCH screening and treatment trials (NCT03155620 and NCT03213704) are similarly studying the effectiveness of treatment directed by genetic testing in pediatric patients with solid tumors, non-Hodgkin lymphomas, or histiocytic disorders that have either progressed following at least one line of standard systemic therapy and/or for which no standard treatment exists that has been shown to prolong survival. Another phase II trial (NCT03834961) is evaluating the efficacy and safety of neoadjuvant larotrectinib in pediatric patients with previously untreated TRK fusion solid tumors (including infantile fibrosarcoma) or relapsed TRK fusion acute leukemia. A study with more than 15 years’ follow-up of childhood cancer survivors who received larotrectinib will also be needed to determine if there is a potential longer-term risk of adverse effects of larotrectinib on growth and development.

## Conclusion

The approval of larotrectinib in more than 40 countries marks not only a milestone in its development but also in the development of tumor-agnostic approvals. Ongoing clinical trials have shown positive efficacy and safety results. The aim of the ON-TRK non-interventional study is to investigate the effects of larotrectinib in a broader patient population in real-world clinical practice conditions and determine the impact of larotrectinib use on development in pediatric patients followed for at least 5 years from the initiation of larotrectinib.

## Data Availability

Not applicable.

## Abbreviations

AE: Adverse event
CI: Confidence interval
CNS: Central nervous system
CTCAE: Common Terminology Criteria for Adverse Events
DCR: Disease control rate
DOR: Duration of response
EMA: European Medicines Agency
FDA: US Food and Drug Administration
FISH: Fluorescence in situ hybridization
GVP: Good pharmacovigilance practices
IHC: Immunohistochemistry
IRC: Independent review committee
MedDRA: Medical Dictionary for Regulatory Activities
NCI: National Cancer Institute
NGS: Next-generation sequencing
*NTRK*: Neurotrophic tyrosine receptor kinase
ORR: Objective response rate
OS: Overall survival
PFS: Progression-free survival
RT-PCR: Reverse-transcription polymerase chain reaction
SAE: Serious adverse event
TEAE: Treatment-emergent adverse event
TRK: Tropomyosin receptor kinase
TTR: Time to response
